# Renal Vascular Involvement Assessed by Intrarenal Resistive Index in Patients with Rheumatoid Arthritis: Associations with Structural Joint Damage and Cardiovascular Risk

**DOI:** 10.3390/jcm15051991

**Published:** 2026-03-05

**Authors:** Alexandru Caraba, Deiana Roman, Mircea Iurciuc, Stela Iurciuc

**Affiliations:** 1 University Clinic of Internal Medicine—Diabetes, Nutrition, Metabolic Diseases, and Systemic Rheumatology, “Victor Babes” University of Medicine and Pharmacy Timisoara, 300041 Timișoara, Romania; caraba.alexandru@umft.ro; 2 Railway Clinical Hospital Timișoara, 300173 Timișoara, Romania; 3 Cardiology Department, “Victor Babes” University of Medicine and Pharmacy Timisoara, 300041 Timișoara, Romania; iurciuc.mircea@umft.ro; 4 Internal Medicine and Ambulatory Care, Prevention and Cardiovascular Recovery Discipline, “Victor Babes” University of Medicine and Pharmacy Timisoara, 300041 Timișoara, Romania; iurciuc.stela@umft.ro

**Keywords:** rheumatoid arthritis, chronic kidney disease, renal resistive index, cardiovascular risk, structural joint damage

## Abstract

**Background/Objectives**: Patients with rheumatoid arthritis (RA) have an increased risk of chronic kidney disease (CKD) and cardiovascular disease, largely driven by persistent systemic inflammation. This study aimed to assess the risk of CKD in RA patients and to evaluate its association with structural joint damage and cardiovascular risk (CVR). **Methods**: In this cross-sectional study, 70 patients fulfilling the 2010 ACR/EULAR criteria for RA were evaluated. Structural joint damage was assessed using the Sharp/van der Heijde score (SHS). Renal involvement was evaluated by estimated glomerular filtration rate (eGFR), urinary albumin-to-creatinine ratio (ACR), and intrarenal resistive index (RRI). CVR was assessed using the SCORE system, adjusted according to EULAR recommendations, and carotid ultrasonography was performed to assess intima–media thickness (IMT) and atherosclerotic plaques. **Results**: SHS was significantly correlated with renal and vascular parameters, showing positive associations with ACR, RRI, and carotid IMT, and a negative correlation with eGFR (all *p* < 0.0001). CVR correlated positively with SHS, ACR, RRI, and IMT. Patients with elevated RRI (≥0.70) had longer disease duration, more severe joint damage, impaired renal function, and higher CVR. **Conclusions**: In RA patients, cumulative articular damage is closely associated with renal dysfunction and increased CVR, highlighting the central role of sustained inflammation in multiorgan involvement.

## 1. Introduction

Rheumatoid arthritis (RA) represents the most frequent rheumatological disorder characterized by chronic inflammation, the primary target being the synovial membrane of the joints [1]. In absence of proper treatment, it has a progressive evolution involving articular damage, alongside extra-muscular-skeletal manifestations, having a negative impact on the life quality of these patients [2]. Among the extraarticular manifestations, interstitial lung disease, cardiovascular and renal complications carry significant importance [3,4]. While interstitial lung disease is one of the major prognostic factors for patients with RA, it is well known that patients with RA have a higher cardiovascular risk (CVR), underlining the importance of early diagnosis and comprehensive treatment strategies [2]. Furthermore, there is a well-established interdependence between the heart and the kidney, rendered of even higher importance in patients with RA, in which the risk of chronic kidney disease (CKD) is increased.

Renal involvement, one of the most important systemic complication of RA poses significant diagnostic and management issues [5,6]. There is an increasing body of evidence reporting a higher incidence and prevalence of renal dysfunction among RA patients. The discordance in data can be explained by the criteria which were used to define renal dysfunction [7,8,9,10]. The etiology of renal involvement in RA patients remain incompletely elucidated and controversial even at present [11]. In RA, renal disease may be associated with the effects of the primary disease process, as well as with the side effects of the medication used in its treatment.

Renal biopsy findings in patients with rheumatoid arthritis (RA) have demonstrated a wide spectrum of histopathological lesions. The most frequently reported abnormalities include glomerular diseases such as membranous nephropathy, mesangial proliferative glomerulonephritis, IgA nephropathy, minimal change disease, focal segmental glomerulosclerosis, and secondary AA amyloidosis. Additional findings include tubulointerstitial nephritis, acute tubular necrosis, papillary necrosis, and rheumatoid vasculitis [4]. The pattern of renal involvement in RA has changed substantially with the introduction of biologic and targeted synthetic disease-modifying antirheumatic drugs (DMARDs). Earlier therapeutic agents, including penicillamine, gold salts, and cyclosporine, were associated with a higher risk of nephrotoxicity. In contrast, modern targeted therapies have resulted in improved disease control, reduced drug-induced renal injury, and a marked decline in the incidence of secondary AA amyloidosis [11].

Despite these advances, patients with RA remain at increased risk of developing (CKD), independent of overt renal pathology. Multiple factors contribute to CKD development in this population. Chronic systemic inflammation and mitochondrial dysfunction are recognized as key mechanisms promoting renal fibrosis and progressive kidney damage [12,13], being associated with the decline in estimated glomerular filtration rate (eGFR) [14]. Kochi et al. were among the first to demonstrate that persistent inflammation lasting at least six months in patients with RA is associated with an increased risk of CKD [13]. Sustained elevations of proinflammatory cytokines, particularly tumor necrosis factor-alpha (TNF-α) and interleukin-6 (IL-6), have been identified as predictors of renal function decline [15]. Long-standing systemic inflammation may exert direct deleterious effects on the kidney through multiple pathophysiological pathways, including endothelial dysfunction, oxidative stress, and fibrotic remodeling [13].

The intrarenal resistive index (RRI) is a Doppler ultrasonography parameter used as a marker of renal vascular resistance, with normal values generally ≤0.70 [16]. Elevated RRI values in native kidneys have been associated with impaired renal function, increased renal parenchymal damage, and a higher incidence of adverse cardiovascular events [17].

Chronic inflammation in RA is also strongly linked to accelerated atherosclerosis, contributing to increased cardiovascular morbidity and mortality [2]. A close bidirectional relationship exists between cardiovascular disease and chronic kidney disease, with declining renal function closely correlating with an increased CVR [18].

The aim of this study is to assess the risk of CKD in patients with rheumatoid arthritis and to investigate its association with articular damage and CVR. Particular emphasis was placed on the RRI as a marker of renal vascular involvement and its potential added value over conventional renal and CVR markers.

## 2. Materials and Methods

The present cross-sectional study included 70 patients with that were admitted to the Department of Rheumatology within the Railway Clinical Hospital in Timișoara, Romania, between August 2019 and August 2025. All participants fulfilled the 2010 American College of Rheumatology/European Alliance of Associations for Rheumatology classification criteria for RA [19]. Exclusion criteria were as follows: age < 18 years; unable to give informed consent; presence of overlap connective tissue diseases; a prior diagnosis of CKD, including glomerulonephritis, tubulointerstitial nephritis, vascular nephropathy, or AA amyloidosis; significant proteinuria (>500 mg/g creatinine); a preexisting diagnosis of diabetes mellitus or hypertension; eGFR < 30 mL/min/1.73 m^2^; pregnancy or breastfeeding; and active smoker status. All patients provided written informed consent prior to enrollment. The study was conducted in accordance with the Declaration of Helsinki and was approved by the Ethics Committee of the Railway Clinical Hospital Timișoara, Romania (approval number 62, 12 September 2018).

Medical history and current medication use were recorded for all participants. A complete clinical examination was performed in all patients.

Laboratory assessments included rheumatoid factor (RF), measured by immunonephelometry, and anti–citrullinated protein antibodies (ACPA), assessed using a chemiluminescent microparticle immunoassay (CMIA). Serum total cholesterol and high-density lipoprotein (HDL) cholesterol levels were measured by spectrophotometric methods in all patients.

Structural joint damage was evaluated using the SHS. This validated scoring system assesses the extent of radiographic joint damage—specifically bone erosions and joint space narrowing—on conventional radiographs of the hands and feet. Bone erosions were scored on a scale from 0 to 5 per joint, while joint space narrowing was scored from 0 to 4 per joint. The total SHS ranges from 0 (no damage) to 448 (maximum structural damage) [20]. Joint damage was assessed at study inclusion.

Renal involvement was assessed using the urinary albumin-to-creatinine ratio (ACR), eGFR, and RRI.

For urinary ACR determination, a first-morning urine sample was collected. Urinary albumin concentration was measured by immunonephelometry and urinary creatinine by an enzymatic method. The ACR was calculated as the ratio of albumin to creatinine and expressed as milligrams of albumin per gram of creatinine (mg/g). According to KDIGO guidelines, ACR values were classified as normal (<30 mg/g), moderately increased albuminuria (30–300 mg/g), or severely increased albuminuria (>300 mg/g) [21].

Estimated glomerular filtration rate was calculated using the CKD Epidemiology Collaboration (CKD-EPI) 2021 creatinine-based equation, based on serum creatinine levels.

Renal ultrasonography was performed in all participants using an ARIETTA 65 ultrasound system (Hitachi Ltd., Tokyo, Japan) equipped with a 3.5 MHz convex transducer, by two experienced operators (A.C. and M.I.). Examinations were conducted after a minimum fasting period of six hours. RRI measurements were obtained from the interlobar arteries at the upper, mid, and lower poles of each kidney, with patients examined in the lateral decubitus or prone position. Color Doppler gain and pulse repetition frequency were adjusted individually to avoid aliasing. The Doppler angle was maintained below 60° relative to the long axis of the vessel. The mean RRI value from both kidneys was calculated for analysis. An RRI value < 0.70 was considered normal [22,23].

Vascular involvement was evaluated using carotid ultrasonography, assessing carotid IMT and the presence of carotid plaques. Examinations were performed using the ARIETTA 65 ultrasound system with a 5 MHz linear transducer by the same two operators. IMT was measured on the far wall of the common carotid artery, approximately 2 cm proximal to the carotid bifurcation. A focal wall thickening > 1.5 mm was defined as a carotid plaque [24].

CVR was assessed using the SCORE system for high-risk European populations [25]. In accordance with EULAR recommendations, the calculated SCORE value was multiplied by 1.5 in patients with rheumatoid arthritis duration > 10 years, seropositivity for RF and/or ACPA, or the presence of extra-articular manifestations [26].

### Statistical Analysis

Data was collected and analyzed using SPSS v.17 statistical software package (SPSS Inc., Chicago, IL, USA). Data distribution was assessed using the Kolmogorov–Smirnov test. Normally distributed variables were expressed as mean ± standard deviation. Interobserver reliability for SHS assessment was evaluated using the intraclass correlation coefficient (ICC). Comparisons and associations were analyzed using parametric statistical methods, including analysis of variance (ANOVA) and Pearson’s correlation coefficient. A *p* value < 0.05 was considered statistically significant.

## 3. Results

The present study included 70 patients of which 58 (82.85%) were female and 12 (17.14%) were male. The median age of the patients was 59.48 ± 6.54 and the median disease duration was 15.38 ± 6.56. The main characteristics of the patients with RA enrolled in this study are presented in Table 1.

Although no a priori sample size calculation was performed, a post hoc power analysis indicates that with 70 participants, the study had >99% power to detect correlations of the magnitude observed (r ≥ 0.70) at a two-sided α level of 0.05.

All patients with RA included in the study were seropositive. The mean rheumatoid factor (RF) level was 212.76 ± 65.92 IU/mL, and the mean anti–citrullinated protein antibody (ACPA) level was 193.53 ± 62.81 IU/mL.

Treatment in the studied lot included Methotrexate and Leflunomide either as monotherapy (in 28 and 6 patients, respectively), or associated with anti TNF-α medication (in 20 and 16 patients, respectively). No other biological treatment was utilized in the studied lot. Corticoid therapy was utilized only in 12 cases for a duration of 4.72 ± 2.52 weeks after the moment of RA diagnosis. Nonsteroidal anti-inflammatory drugs (NSAIDs) were utilized for pain management as needed.

Renal tubular toxicity associated with NSAID exposure was assessed using the urinary β2-microglobulin–to–creatinine ratio, which showed a mean value of 93.44 ± 28.74 μg/g, indicating subclinical tubular involvement. According to the Steinbrocker radiographic classification, patients were distributed as follows: stage I (n = 4), stage II (n = 15), stage III (n = 39), and stage IV (n = 12). 

Mean values of the investigated parameters can be found in Table 2.

Chronic inflammatory burden in rheumatoid arthritis is reflected by the extent of structural joint damage, as quantified by the SHS. Interobserver agreement for SHS was excellent, with an ICC of 0.9889. Sustained systemic inflammation is also associated with vascular involvement affecting both the renal and carotid arterial beds. Renal vascular involvement was assessed using the RRI, while carotid artery involvement was evaluated by measuring IMT and identifying the presence of carotid atherosclerotic plaques.

Significant associations were identified between articular damage and renal and vascular parameters. SHS showed statistically significant positive correlations with urinary ACR, RRI, and carotid IMT, and a statistically significant negative correlation with eGFR. These correlations are presented in Table 3.

**Figure 1:**
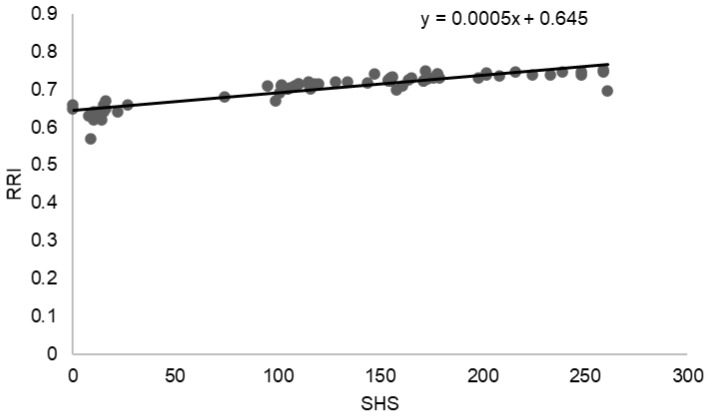
Correlation between SHS and RRI.

A strong positive association was observed between structural joint damage and renal vascular involvement. As illustrated in Figure 1, the SHS was positively correlated with the RRI, indicating higher renal vascular resistance with increasing articular damage. Linear regression analysis demonstrated a direct relationship between SHS and RRI (y = 0.0005x + 0.645), consistent with the strong correlation identified in the overall analysis (r = 0.8794, *p* < 0.0001). Patients with higher SHS values tended to exhibit elevated RRI measurements, suggesting an association between cumulative inflammatory joint damage and renal vascular impairment.

**Figure 2:**
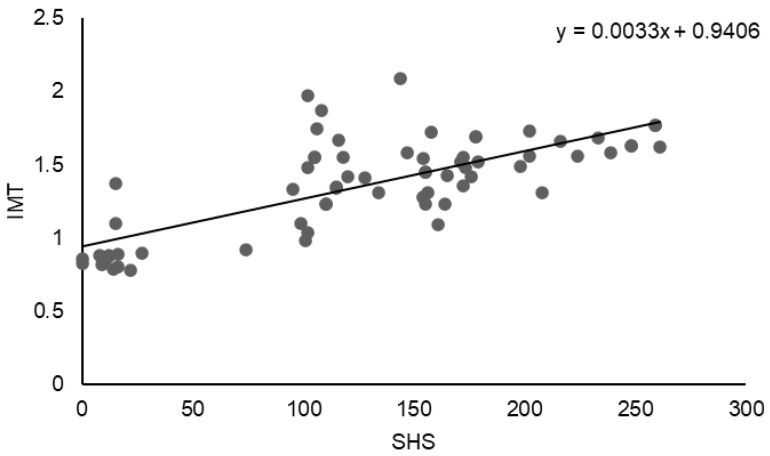
Correlation between SHS and carotid IMT.

A positive association was also observed between structural joint damage and carotid arterial involvement. As shown in Figure 2, the SHS was positively correlated with carotid IMT. Linear regression analysis demonstrated a direct relationship between SHS and IMT (y = 0.0033x + 0.9406), indicating increased carotid wall thickness with higher degrees of radiographic joint damage. This finding is consistent with the statistically significant correlation identified in the overall analysis (r = 0.7451, *p* < 0.0001), suggesting that greater cumulative articular damage is associated with increased subclinical atherosclerosis.

CVR was significantly associated with both articular damage and renal and vascular parameters. The CVR score (CV-R) demonstrated a strong positive correlation with the SHS (r = 0.7493, *p* < 0.0001). Significant positive correlations were also observed between CV-R and ACR (r = 0.7014, *p* < 0.0001), RRI (r = 0.7794, *p* < 0.0001), and carotid IMT (r = 0.7588, *p* < 0.0001). These results are summarized in Table 4.

**Figure 3:**
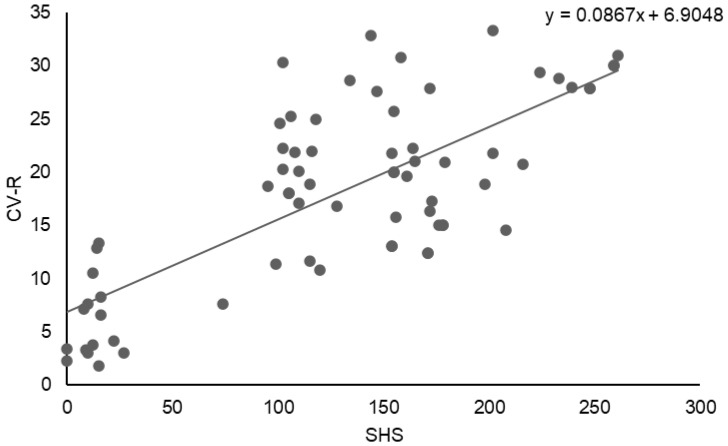
Correlation between SHS and CVR.

A strong positive association was observed between structural joint damage and CVR. As illustrated in Figure 3, the SHS showed a significant positive correlation with CVR (CV-R). Linear regression analysis demonstrated a direct relationship between SHS and CV-R (y = 0.0867x + 6.9048), indicating progressively higher CVR with increasing radiographic joint damage. This finding is consistent with the correlation analysis, which revealed a strong positive association between SHS and CV-R (r = 0.7493, *p* < 0.0001).

To further identify independent determinants of CVR, renal damage, and renal vascular resistance, multivariable regression analyses were performed.

Multivariable regression analyses were performed to identify independent predictors of CVR, ACR, and RRI. CVR was independently associated with age (*p* < 0.0001), disease duration (*p* < 0.001), ACR (*p* < 0.05), and the SHS (*p* < 0.05), according to the following model:

CVR = −33.63 + 0.72 × age + 0.51 × disease duration − 0.02 × ACR + 0.02 × SHS.

ACR was independently associated with disease duration (*p* < 0.0001), SHS (*p* < 0.001), and age (*p* < 0.05), but not with total cholesterol levels, as described by the model:

ACR = −0.65 − 0.14 × age + 3.19 × disease duration + 0.06 × SHS + 0.06 × total cholesterol.

RRI was independently associated with SHS (*p* < 0.0001), disease duration (*p* < 0.001), total cholesterol (*p* < 0.001), ACR (*p* < 0.05), and age (*p* < 0.05), according to the regression model:

RRI = 0.62 − 0.0005 × age + 0.0024 × disease duration + 0.0003 × SHS + 0.0002 × total cholesterol − 0.0001 × ACR.

In addition, a strong positive correlation was observed between RRI and CVR (r = 0.7014, *p* < 0.0001). Based on the established normal cutoff value of RRI < 0.70, patients were categorized into two groups: those with normal RRI values and those with elevated RRI values. The clinical, laboratory, and imaging parameters of the two rheumatoid arthritis patient groups are presented in Table 5.

Patients with elevated RRI ≥ 0.70 differed significantly from those with normal RRI values across multiple demographic, clinical, renal, and cardiovascular parameters. While no significant difference was observed in sex distribution between the two groups, patients with elevated RRI were significantly older (61.69 ± 4.66 vs. 53.11 ± 7.10 years, *p* < 0.0001) and had a markedly longer disease duration (18.59 ± 3.60 vs. 6.11 ± 3.61 years, *p* < 0.0001).

Structural joint damage was substantially more severe in the elevated RRI group, as reflected by significantly higher SHS (163.07 ± 47.59 vs. 25.55 ± 31.36, *p* < 0.0001). Renal function was also significantly impaired in patients with elevated RRI, who exhibited lower eGFR values (58.55 ± 10.98 vs. 86.27 ± 8.71 mL/min/1.73 m^2^, *p* < 0.0001) and higher ACR levels (72.79 ± 20.40 vs. 22.41 ± 7.06 mg/g, *p* < 0.0001).

In addition, patients with elevated RRI demonstrated a significantly higher CVR score (21.60 ± 6.18% vs. 7.49 ± 5.63%, *p* < 0.0001), higher total cholesterol levels (208.19 ± 25.37 vs. 180.00 ± 19.65 mg/dL, *p* < 0.0001), and lower high-density lipoprotein cholesterol levels (47.40 ± 5.06 vs. 60.55 ± 7.25 mg/dL, *p* < 0.0001). Markers of subclinical atherosclerosis were also more pronounced in the elevated RRI group, with significantly increased carotid IMT (1.50 ± 0.21 vs. 0.91 ± 0.14 mm, *p* < 0.0001) and a higher prevalence of carotid atherosclerotic plaques (57.69% vs. 5.55%, *p* < 0.0001).

CVR demonstrated a statistically significant association with SHS, carotid IMT, RRI, and ACR. Patients with rheumatoid arthritis (RA) exhibiting elevated RRI had a significantly higher CVR compared with those with normal RRI values (*p* < 0.0001). This subgroup was characterized by significantly higher total cholesterol levels and significantly lower high-density lipoprotein cholesterol concentrations (*p* < 0.0001). The observed increase in CVR appears to be driven by the combined effects of chronic RA-related systemic inflammation and concomitant renal dysfunction.

## 4. Discussion

In this cross-sectional cohort, patients with rheumatoid arthritis exhibited a high burden of renal and cardiovascular abnormalities that were consistently associated with the severity of structural joint damage. These findings reinforce the concept that articular destruction, renal impairment, and CVR frequently coexist in patients with long-standing RA.

Chronic systemic inflammation has been widely proposed as a shared biological mechanism underlying joint damage, renal abnormalities, and CVR in rheumatoid arthritis [27,28]. Pro-inflammatory cytokines such as tumor necrosis factor-α, interleukin-1, and interleukin-6 are implicated not only in synovial inflammation and radiographic progression, but also in endothelial dysfunction, vascular remodeling, and renal injury [29]. Elevated circulating and urinary levels of these mediators have been reported in patients with chronic kidney disease, supporting their potential contribution to renal involvement in RA [30].

Previous studies have demonstrated that persistent inflammatory activity is associated with accelerated radiographic progression in RA. Elevated multibiomarker disease activity scores have been shown to predict subsequent increases in SHS, particularly in patients with sustained inflammation. Additional observational data suggest that older age, swollen joint counts, and prolonged disease duration further contribute to more rapid structural joint damage. Patients with high MBDA scores exhibited a significantly greater risk of SHS progression over the subsequent year compared with those with moderate or low MBDA scores (RR 3.738; 95% CI 1.448–9.655). In contrast, moderate MBDA scores were not associated with a significantly increased risk of progression compared with low scores (RR 1.437; 95% CI 0.454–4.545) [31]. Similarly, Abacar et al. reported that persistent inflammation was significantly associated with accelerated radiographic progression (*p* < 0.025), while older age (*p* < 0.017) and swollen joint count (*p* < 0.009) were independently associated with rapid SHS progression [32].

Renal abnormalities are increasingly recognized in RA populations. Large observational cohorts have reported a substantial prevalence of reduced estimated glomerular filtration rate, proteinuria, and urinary sediment abnormalities [33]. Meta-analytic data further indicate that RA is associated with a significantly higher risk of developing chronic kidney disease compared with the general population, underscoring the clinical relevance of systematic renal assessment in these patients [9,10,11]. A meta-analysis by Raksasuk and Ungprasert showed that patients with RA have a 52% higher risk of developing CKD compared with the general population [34].

Experimental and clinical evidence suggests that inflammatory cytokines may influence renal microvascular function and structural integrity. Tumor necrosis factor-α has been associated with intrarenal vasoconstriction, inflammatory infiltration, and fibrotic changes, while interleukin-6 and interleukin-1 have been linked to glomerular and tubular injury. These mechanisms may contribute to the observed associations between reduced renal function, albuminuria, and adverse cardiovascular outcomes in RA., with elevated levels being associated with reduced eGFR and increased cardiovascular events. IL-6 contributes to glomerular injury involving podocytes and mesangial cells, as well as tubular epithelial damage, and is linked to systemic inflammation, anemia, and increased cardiovascular mortality. IL-1 induces renal inflammation, podocyte loss, and adverse cardiovascular outcomes [30]. Renal involvement in RA typically follows a progressive course, characterized by a decline in the number of functioning nephrons and advancing nephrosclerosis [13,21]. Regardless of etiology, renal dysfunction constitutes an independent predictor of morbidity and mortality in RA and is strongly associated with CVR [12].

The RRI reflects renal vascular resistance and arterial stiffness, capturing functional and structural alterations that may not be fully reflected by estimated glomerular filtration rate or albuminuria alone. In the present study, RRI demonstrated strong associations with cumulative articular damage, lipid abnormalities, albuminuria, and CVR. Importantly, RRI effectively differentiated patients with more advanced systemic involvement, suggesting that it may provide complementary information beyond conventional renal and cardiovascular markers in RA.

The RRI, measured at the level of the interlobar arteries, reflects the presence of tubulointerstitial and vascular lesions and correlates most strongly with renal arteriolosclerosis and histological chronicity. Elevated RRI values are associated with CKD progression, reduced eGFR, albuminuria, proteinuria, hypertension, and increased CVR [35]. Increased RRI, a marker of intrarenal arterial stiffness, has also been associated with elevated high-sensitivity C-reactive protein levels, further supporting its link to systemic inflammation [36].

In the present study, SHS—an indicator of cumulative joint damage and persistent inflammation—was positively and significantly correlated with ACR (r = 0.7985, *p* < 0.0001), RRI (r = 0.8794, *p* < 0.0001), and carotid IMT (r = 0.7451, *p* < 0.0001), and negatively correlated with eGFR (r = −0.7390, *p* < 0.0001). Hickson et al. reported that elevated erythrocyte sedimentation rate in RA was associated with an increased risk of both CKD (eGFR < 60 mL/min/1.73 m^2^) and cardiovascular disease (HR 1.93; 95% CI 1.04–3.58; *p* = 0.04) [9]. In a large cohort of non-diabetic subjects, Stuveling et al. demonstrated that elevated C-reactive protein levels were positively associated with cardiovascular and renal risk factors, including increased urinary albumin excretion and reduced eGFR (OR 1.8; 95% CI 1.2–2.6) [37].

Verma et al. found a significantly higher prevalence of microalbuminuria in RA patients compared with controls (26% vs. 4%), with correlations to inflammatory markers, disease duration, and joint involvement, suggesting that microalbuminuria reflects systemic inflammatory burden [38]. Panoulas et al. identified microalbuminuria in 12% of RA patients and proposed a link between elevated TNF-α levels and multiorgan damage, including cardiac and renal involvement [39]. Sihvonen et al. demonstrated that microalbuminuria predicted increased cardiovascular mortality in RA (HR 2.77) [5]. Pedersen et al. observed microalbuminuria in 27.7% of RA patients versus 7.8% of controls, with significant associations with inflammatory markers and disease duration [40].

Using a threshold RRI value of 0.70, patients were stratified into two groups. RA patients with RRI < 0.70 were normoalbuminuric (urinary ACR 22.41 ± 7.06 mg/g) and exhibited preserved renal function (eGFR 86.27 ± 8.71 mL/min). In contrast, patients with RRI ≥ 0.70 were significantly older, had longer disease duration, higher SHS, increased urinary ACR, and reduced eGFR (all *p* < 0.0001). Consistent with the close interaction between renal dysfunction and cardiovascular disease, these patients also exhibited increased carotid IMT and a higher prevalence of atherosclerotic carotid plaques (both *p* < 0.0001).

Patients with long-standing active RA also demonstrated adverse lipid profiles, characterized by reduced HDL-cholesterol and elevated total cholesterol levels, favoring the development of premature atherosclerosis [41].

The present study has certain limitations, including its cross-sectional design, which precludes causal inference, and the absence of an a priori sample size calculation. Nonetheless, the relatively large effect sizes observed indicate that the study was adequately powered to detect clinically meaningful associations. Although conducted at a single center with a moderate sample size, the study is strengthened by the comprehensive and simultaneous evaluation of structural joint damage, renal function, renal vascular resistance, and subclinical atherosclerosis in a well-characterized rheumatoid arthritis cohort. The use of the SHS and RRI represents a distinctive aspect of this work, allowing integrated assessment of cumulative inflammatory burden and renal vascular involvement. These features support the robustness and clinical relevance of the findings while underscoring the need for longitudinal, multicenter studies to confirm and extend the observed associations.

Collectively, these findings support a close association between sustained inflammatory burden, joint destruction, renal impairment, and cardiovascular involvement in RA. This study has certain limitations, including its single-centre design and the inclusion of patients at different radiographic stages. Additionally, the use of the MDRD formula for eGFR estimation may underestimate true renal function in some RA patients due to reduced muscle mass and lower creatinine production.

## 5. Conclusions

In patients with rheumatoid arthritis, greater cumulative structural joint damage is associated with impaired renal function and increased cardiovascular risk. These findings highlight the close interrelationship between articular destruction, renal vascular involvement, and cardiovascular burden in RA and support the potential value of integrated, multidisciplinary risk assessment.

## Figures and Tables

**Table 1:** Patient lot characteristics.

Parameter	Value
Total number of patients	70
Women ^a^	58 (82.85%)
Men ^a^	12 (17.14%)
Age ^b^ (years)	59.48 ± 6.54
Disease duration ^b^ (years)	15.38 ± 6.56
DAS-28 ^b^	4.71 ± 4.04
CRP ^b^ (mg/L)	28.05 ± 19.82
Medication used by patients with RA	
Methotrexate	26
Leflunomide	8
Methotrexate + biologic treatment	20
Leflunomide + biologic treatment	16

^a^ Categorical variables. Results are presented as number of cases and (percentage from the total); ^b^ Numerical variables with Gaussian distribution. Results are presented as mean ± standard deviation.

**Table 2:** Investigated parameters.

Parameter	Value
SHS ^b^	127.71 ± 74.71
eGFR ^b^ (mL/min)	65.68 ± 16.02
Urinary ACR ^b^ (mg/g)	59.84 ± 28.49
RRI ^b^	0.70 ± 0.03
CVR ^b^ (%)	17.97 ± 8.64
Total cholesterol ^b^ (mg/dL)	200.94 ± 26.93
HDL-cholesterol ^b^ (mg/dL)	50.78 ± 8.08
Carotid IMT ^b^ (mm)	1.35 ± 0.32
Carotid atherosclerotic plaques ^a^	Present in 31 patients (44.28%)

^a^ Categorical variables. Results are presented as number of cases and (percentage from the total); ^b^ Numerical variables with Gaussian distribution. Results are presented as mean ± standard deviation.

**Table 3:** Correlations between SHS and kidney and carotid arteries involvement.

Correlation Between SHS and	r	*p*
eGFR	−0.7390	<0.0001
ACR	0.7985	<0.0001
RRI	0.8794	<0.0001
Carotid IMT	0.7451	<0.0001

eGFR = estimated glomerular filtration rate; ACR = urinary albumin-to-creatinine ratio; RRI = intrarenal resistive index; Carotid IMT = carotid intima–media thickness.

**Table 4:** Correlations between CVR and the studied parameters.

Correlation Between CV-R and:	r	*p*
SHS	0.7493	<0.0001
Urinary ACR	0.7014	<0.0001
RRI	0.7794	<0.0001
Carotid IMT	0.7588	<0.0001

**Table 5:** Differences between patients with normal RRI values vs. patients with elevated RRI values.

Parameter	RRI < 0.70	RRI ≥ 0.70	*p*
Patients			
men	5	7	>0.05
women	13	45	<0.001
Mean age (years)	53.11 ± 7.10	61.69 ± 4.66	<0.0001
Disease duration of evolution (years)	6.11 ± 3.61	18.59 ± 3.60	<0.0001
SHS	25.55 ± 31.36	163.07 ± 47.59	<0.0001
eGFR (mL/min)	86.27 ± 8.71	58.55 ± 10.98	<0.0001
Urinary ACR (mg/g)	22.41 ± 7.06	72.79 ± 20.40	<0.0001
CVR (%)	7.49 ± 5.63	21.60 ± 6.18	<0.0001
Total cholesterol (mg/dL)	180 ± 19.65	208.19 ± 25.37	<0.0001
HDL-cholesterol (mg/dL)	60.55 ± 7.25	47.40 ± 5.06	<0.0001
Carotid IMT (mm)	0.91 ± 0.14	1.50 ± 0.21	<0.0001
Carotid atherosclerotic plaques	5.55%	57.69%	<0.0001

## Data Availability

The original contributions presented in this study are included in the article. Further inquiries can be directed to the corresponding author.

## Abbreviations

The following abbreviations are used in this manuscript:

RA	Rheumatoid arthritis
CKD	Chronic kidney disease
eGFR	Estimated glomerular filtration rate
RRI	Intrarenal resistive index
IMT	Intima media thickness
SHS	Sharp/van der Heijde score
CVR	Cardiovascular risk
ICC	Intraclass correlation coefficient
TNF-α	Tumor necrosis factor α
IL-6	Interleukin 6
